# DETEXA: declarative extensible text exploration and analysis through SQL

**DOI:** 10.1007/s00799-023-00358-1

**Published:** 2023-05-10

**Authors:** Yannis Foufoulas, Eleni Zacharia, Harry Dimitropoulos, Natalia Manola, Yannis Ioannidis

**Affiliations:** 1 Department of Informatics and Telecommunications, National and Kapodistrian University of Athens, Panepistimiopolis, 15784 Ilisia, Greece; 2 Athena Research Center, Artemidos 6 & Epidavrou, 15125 Marousi, Greece

**Keywords:** Text analytics, YeSQL, User-defined functions

## Abstract

Metadata enrichment through text mining techniques is becoming one of the most significant tasks in digital libraries. Due to the exponential increase of open access publications, several new challenges have emerged. Raw data are usually big, unstructured, and come from heterogeneous data sources. In this paper, we introduce a text analysis framework implemented in extended SQL that exploits the scalability characteristics of modern database management systems. The purpose of this framework is to provide the opportunity to build performant end-to-end text mining pipelines which include data harvesting, cleaning, processing, and text analysis at once. SQL is selected due to its declarative nature which offers fast experimentation and the ability to build APIs so that domain experts can edit text mining workflows via easy-to-use graphical interfaces. Our experimental analysis demonstrates that the proposed framework is very effective and achieves significant speedup, up to three times faster, in common use cases compared to other popular approaches.

## Introduction

The exponential growth of published articles opens up new challenges and research opportunities. Text mining has gained significant attention across a broad range of applications. The researchers implement text mining workflows to enhance the understanding of academic literature and generate new knowledge. With text mining on scientific literature, it is possible to identify and classify the key themes of an academic field, explore trends, assess the impact and popularity of topics over a period of time, help authors to find published literature related to their research, and so on.

Python is the language that many data scientists prefer for text analysis as it is easy to learn and enhances their productivity, given its modules for text and data mining tasks (e.g., NLTK [1]). However, NLTK is usually sub-optimal in terms of performance, as Python is designed to be an easy-to-use high-level dynamic language. Scalable analytical frameworks that support Python are often used to make Python text analytics run faster (e.g., PySpark [2], Dask [3]). Database management systems (DBMSs) also support the execution of user-defined functions (UDFs) written in Python [4–6]. However, these works come with several limitations, especially for text processing. For example, in PySpark and PostgreSQL, Python functions run in a separate process, introducing big inter-process communication overheads, which is a deterrent factor for text mining that usually involves large texts. Dask targets mainly data analysis on numeric data, as it is based mainly on NumPy and Pandas. Furthermore, UDFs in DBMSs are often sub-optimal in terms of expressiveness, i.e., they are statically typed which contradicts Python’s nature, stateless, and without side effects. Thus, many data scientists do not integrate their text analysis workflows into DBMSs but prefer to use simpler tools like NLTK and PySpark.

In this paper, we present a scalable text analysis framework, DETEXA [7], built on top of YeSQL [8]. Specifically, we have implemented a rich set of text mining functionalities as polymorphic scalar, aggregate, and table UDFs that can work in synergy with various database systems through YeSQL.

We consider YeSQL a perfect match for text analysis tasks as it supports polymorphic and dynamically typed functions which are critical for dealing with various data schemas (i.e., heterogeneous data input JSON, XML, etc.), as is usually the case when harvesting text data from various repositories. Moreover, its performance characteristics fit well with text mining since: 1) it supports stateful UDFs that allow data scientists to run costly operations once at the global scope and reuse them through multiple functions —such operations are usual in text mining (e.g., pattern compilation, external package imports and setups, etc.)—and 2) in-process UDF execution and tracing JIT compilation of Python UDFs enhance the scalability of text mining workflows. Furthermore, the declarative interface of the presented framework, which is inherited by YeSQL, allows quick experimentation concerning data analysis tasks and rapid development of data processing workflows. Finally, it allows the easy implementation of application interfaces, using the most common architecture of the web that consists of a portal frontend and a backend that produces SQL queries and submits them to the DBMS. In OpenAIRE [9], such an interface has been implemented and used by community experts to implement and tune their own information extraction algorithms without requiring any programming knowledge.

The query (shown in Algorithm 1) illustrates the data-intensive part of a classification algorithm, written using the text analysis library of the presented framework.
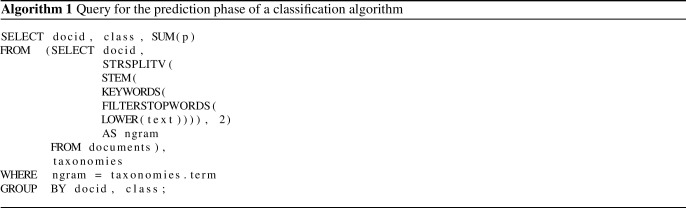


This query reads data from a table with documents and first applies several standard preprocessing steps (i.e., convert to lowercase, stopword removal, tokenization with punctuation removal, and stemming). With the dynamic function STRSPLITV, the input string with tokens is transformed into a database column with n-grams of length up to 2, which is joined against the taxonomies table that consists of the classes, terms, and weights. The latter has already been produced during the offline training phase. Finally, using relational operators the sum-of-term weights per class is calculated. That is, according to the presented framework, the heavy relational parts (i.e., JOIN, GROUP BY, and aggregate operations) are executed by the DBMS, whereas the procedural text processing tasks (preprocessing, pattern matching, etc.) are done with a rich set of Python text analysis functions.

In more detail, the contributions of this work are the following:

*Text analysis library* A rich library of reusable and customizable functions for text mining implemented as scalar, aggregate, and table functions, and executed inside a DBMS.

*Extensibility* Data scientists can implement their own custom functions as scalar, aggregate, or table UDFs, and use them in synergy with the already-supported operations. The functions are implemented in Python files and registered in the underlying database engine.

*Deployment* Currently, DETEXA is deployed and runs in production by OpenAIRE,^1^ a technical infrastructure harvesting research output from connected data providers, which partners up a total of 65 European universities, research centers, and institutions. To date, 146 M publications, 18 M research data records, and 312K research software items, from 111K data sources, linked to 3 M grants from 25 different national funders and 180K organizations, have been harvested using over 150 UDFs. DETEXA has been used in prior works to classify [10] and text mine Open Access publications and extract information like funding information [11], software links, citation links [12], links to datasets and bioentities, etc.

Moreover, contributions of YeSQL, which is our underlying system, allow our text analysis framework to support the following features:

*Declarative language for text mining* Complex text mining algorithms are supported using a declarative language which makes easy the implementation of interactive application interfaces. Such an interface is shown in Sect. 3.4.

*Performance* As the experiments show, the proposed framework outperforms other popular libraries in important text analysis tasks. The text mining field fits well with database UDFs as they are defined in YeSQL, and reaps all its performance benefits.

The structure of the paper is as follows. Section 2 reviews popular works in the field, their advantages and limitations. Section 3 presents an overview of the DETEXA framework and its applicability. Section 4 demonstrates the potential of the presented framework through experiments on well-established algorithms against several popular text mining libraries and systems. Finally, Sect. 5 concludes the paper and discusses potential future directions.

## Related work

In this section, we discuss prior work on text mining with Python, divided into two main categories: (1) Python text mining libraries and (2) Scalable data management systems with Python support. We also touch on deep learning-based models.

### Text analysis tools

The most popular text mining library in Python is NLTK. It is used for tokenization, lemmatization, stemming, parsing, etc. Thanks to its many third-party extensions, it supports many approaches for almost any text mining task. However, it is based on CPython’s interpreter and does not scale well with big data. Other Python libraries for text mining include scikit-learn [13], spaCy [14], Gensim [15], and more. Scikit-learn is a generic tool for machine learning with support for several text analysis tasks. However, since it is built on NumPy it is fast mostly in numeric operations but sub-optimal when processing strings. SpaCy is considered to be faster than NLTK; however, it lacks flexibility as it is not customizable. Gensim was originally developed for topic modeling and is not a complete NLP toolkit like NLTK and SpaCy, thus it should be used in synergy with other libraries. Our work differentiates from these libraries as 1) it is more efficient in terms of performance since it is based on DBMSs, 2) it is simpler to implement new pipelines due to its declarative interface, and 3) it is customizable and extensible.

### Data management systems with text mining opportunities

Several prior efforts have focused on making Python run faster. PySpark is a Python interface to Spark which supports the definition of UDFs written in Python. This is one of the most popular systems for scalable Python among data scientists, which achieves efficiency through parallelization. However, it runs Python UDFs on the interpreter in separate processes, introducing several inter-process communication overheads. Dask is a distributed analytical tool written in Python with Pandas and NumPy. It targets mainly numeric operations and is usually sub-optimal when processing strings: Pandas and NumPy natively support only a few operations on strings, so more complex string analysis is possible only outside NumPy/Pandas which requires string copy transformations into Python Objects. Moreover, several DBMSs support Python UDFs (e.g., PostgreSQL, MonetDB, Vertica, and more). However, they come with several limitations in expressiveness and performance, and thus are rarely the first option for text mining. Specifically, they require UDFs with statically defined schemata which contradicts Python’s dynamic typing, they run Python in a separate process, or they support NumPy which is not designed for string processing. The presented text analysis framework avoids these performance and expressiveness issues due to the polymorphic and dynamic nature of its functions and the performance characteristics introduced by YeSQL.

### Deep learning-based models

Recent works concentrate on new language representation models like BERT [16] which can be used for several tasks like question answering, text classification, and inference. Existing BERT implementations are based on TensorFlow [17] or PyTorch [18]. The presented text analysis framework can be used to implement state-of-the-art techniques like BERT using the already existing UDFs (e.g., data cleaning operators, tokenizers for the creation of the vocabulary), and also using the extensibility features of the framework to implement new functionalities via the supported types of UDFs. Note also that UDFs implemented in DETEXA support the use of popular external Python libraries like PyTorch and integrate already existing code with minimal edits.

## The DETEXA framework

In this section, we analyze the presented framework and its functionalities. Specifically, we present the library of the supported functions, we explain why we selected YeSQL and the opportunities to extend the supported functionalities with fully fledged Python, and finally we present a graphical interface deployed in OpenAIRE which is used by domain experts to run simple information extraction tasks using the presented framework.

### Function library

In order to support end-to-end text analysis pipelines, the framework includes functions in several categories:

- *Data input functions*: polymorphic functions used to process data from heterogeneous sources (i.e, CSV, JSON, XML, PDF and HTML parsers, Web tables, Rest APIs, files in HDFS and storage devices, external database connectors, etc.)
- *Data processing functions*: functions which apply typical text mining steps on input data (i.e., tokenization, stopword removal, etc.)
- *Pattern-matching functions*: a variety of functions for parameterized pattern matching (i.e., pattern extraction, updating, weighted patterns, etc.)
- *Bag-of-words functions*: several functions to support bag-of-words functionalities using JSON ordered or unordered arrays and dictionaries. A JSON array with terms can be converted into a database column and vice versa. A JSON dictionary can be converted into a nested table and used in SQL as any other table.
- *Distance functions*: several functions that calculate distance among JSON arrays (e.g., Jaccard, cosine, edit_distance, and more).
- *Language functions*: statistical functions that process documents to extract language information (e.g., detectlang), or functions that are language specific (e.g., stem).
- *Text filtering functions*: functions which scan a document and return text snippets according to specific patterns or document sections (e.g., textreferences, textabstract, textacknowledgements, textwindow, etc.)
- *Data output functions*: functions which return the result of a text analysis workflow to a permanent location or to standard output in various formats (JSON, CSV, SQLite database, etc.)
- *General purpose functions*: functions that are used for various data handling tasks including sampling, random generators, date functions, mathematical operations, statistics, and more.

Note that the purpose of the framework is not to implement text analysis pipelines using only Python UDFs. This would be sub-optimal as UDFs are considered black boxes for a database optimizer, thus in this case, we could not reap the benefits of in-database execution. However, according to the design of the framework, the relational parts (e.g., join, scan, filter, sort, etc.) of a pipeline are not implemented as part of procedural Python UDFs but are still expressed in the SQL part of a query so that they are executed efficiently by the DBMS, whereas the procedural parts of a pipeline are written in Python UDFs.

All the implemented functions are mapped into scalar, aggregate, or polymorphic table database UDFs according to their scope. Specifically, *data input functions* are implemented as polymorphic table UDFs, as they process external sources and return the input data as database tables. *Data processing*, *pattern matching*, and *language functions* process one row at a time so they are defined as scalars. *Bag-of-words functions* are divided either in aggregates or in scalars returning dynamic schemas, i.e., *jgroup* function processes a group and creates a JSON dictionary or array with all the elements in the group, *jsplit* processes a JSON dictionary or array and splits it into multiple columns, whereas *jsplitv* processes a JSON dictionary or array and splits it vertically into multiple rows. *Distance functions* are implemented as dynamic scalar UDFs that process JSON arrays or plain strings and return their distance. These functions are also implemented as aggregates in case the target data is stored in multiple rows. *Text filtering functions* process one document at a time and return dynamic schemas so they are defined as scalar UDFs. Finally, *data output functions* are implemented as polymorphic table UDFs with side effects, as they export their result table at a user-defined location.

In total, the function library offers more than 150 operators for text analysis. The presented framework with its function library is deployed as a third-party library of OpenAIRE’s Inference Information Service (IIS)^2^ and used by OpenAIRE’s data scientists every day to extract and discover knowledge from OpenAIRE’s publication texts and abstracts, including funding information, bioentities, software links, data citations, document classifications, and more.

### Why YeSQL?

Despite that this work could be integrated into almost any DBMS with Python UDF support, we selected YeSQL as its modularity and expressiveness characteristics make it a perfect fit for the proposed text analysis framework. Specifically, as described in the previous section, YeSQL’s polymorphic and dynamic functions allow the implementation of our reusable data input functions since those functions return dynamic schemata according to the input data. YeSQL’s scalar UDFs returning arbitrary table forms enable the implementation of bag-of-words functions as these functions take one row in their input and return a column or a nested table. YeSQL also allows for UDFs with side effects which fit the nature of data output functions. As for performance, YeSQL’s seamless data transfer of strings between the DBMS and the procedural language which runs in-process is critical for applications on texts. YeSQL’s UDF fusion is a perfect fit with text mining scenarios, as in this context, pipelines of operations are very common (e.g., specifically during preprocessing and data harvesting/transforming). Stateful UDFs also allow for performant pattern matching and data processing functions: external packages are imported and set up at the global layer, and patterns are also precompiled at the global layer and not once per row.

The following query (Algorithm 2) illustrates some of the hallmark functionalities of the proposed framework. It reads raw files containing publication full texts from arXiv and PubMed (in CSV and JSON with two keys containing the *id* and *text*), runs various processing steps (i.e., abstract extraction, tokenization, stopword removal, stemming), creates a JSON array with the top 10% frequent terms, and then calculates the Jaccard similarity. Finally, for each arXiv document, it returns up to 5 PubMed documents (aggregate UDF *top* is similar to native SQL function *max*; however, it returns multiple top N rows) with the highest Jaccard similarity. The analysis result is exported to an external JSON file. An example of the first two rows of such a file is shown below.
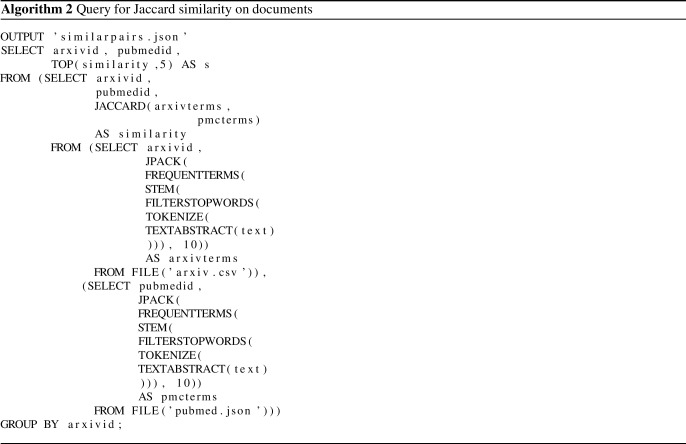

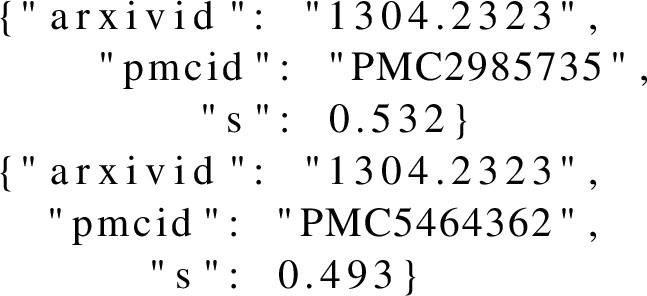


Note that this is not considered to be the best way to calculate document similarity; this could be better based on TF-IDF calculation, but we show this as it covers well some of the main characteristics of the presented framework.

### Extensibility

Although the presented framework comes with a large number of predefined operators, the data scientist is allowed to extend its functionalities by submitting her own custom functions categorized in scalars, aggregates, and polymorphic table functions, according to their scope. These functions are written in Python files with support for external packages. If a function processes one row at a time then this is defined as a scalar. Aggregate functions process a group of rows at a time, and polymorphic table functions process a whole table.

As an example, the function in Algorithm 3 is implemented as a scalar function and converts the input rows into lowercase. However, the following function (Algorithm 4) is implemented as an aggregate UDF and returns a JSON list containing the terms for each group of input rows.
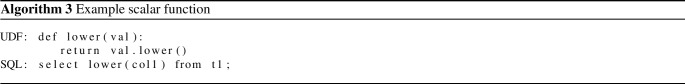

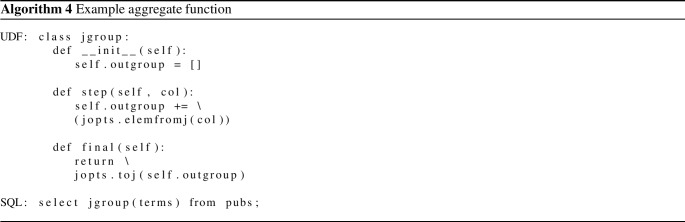


Aggregate UDFs process whole groups of rows incrementally. The function *init* allows for any required initializations, the function *step* is called once per row, and the function *final* produces and returns the final aggregate result.

Similarly, the data scientist is allowed to submit his table-returning functions in Python files and extend the functionalities of the framework.

### Interfaces

**Fig. 1:**
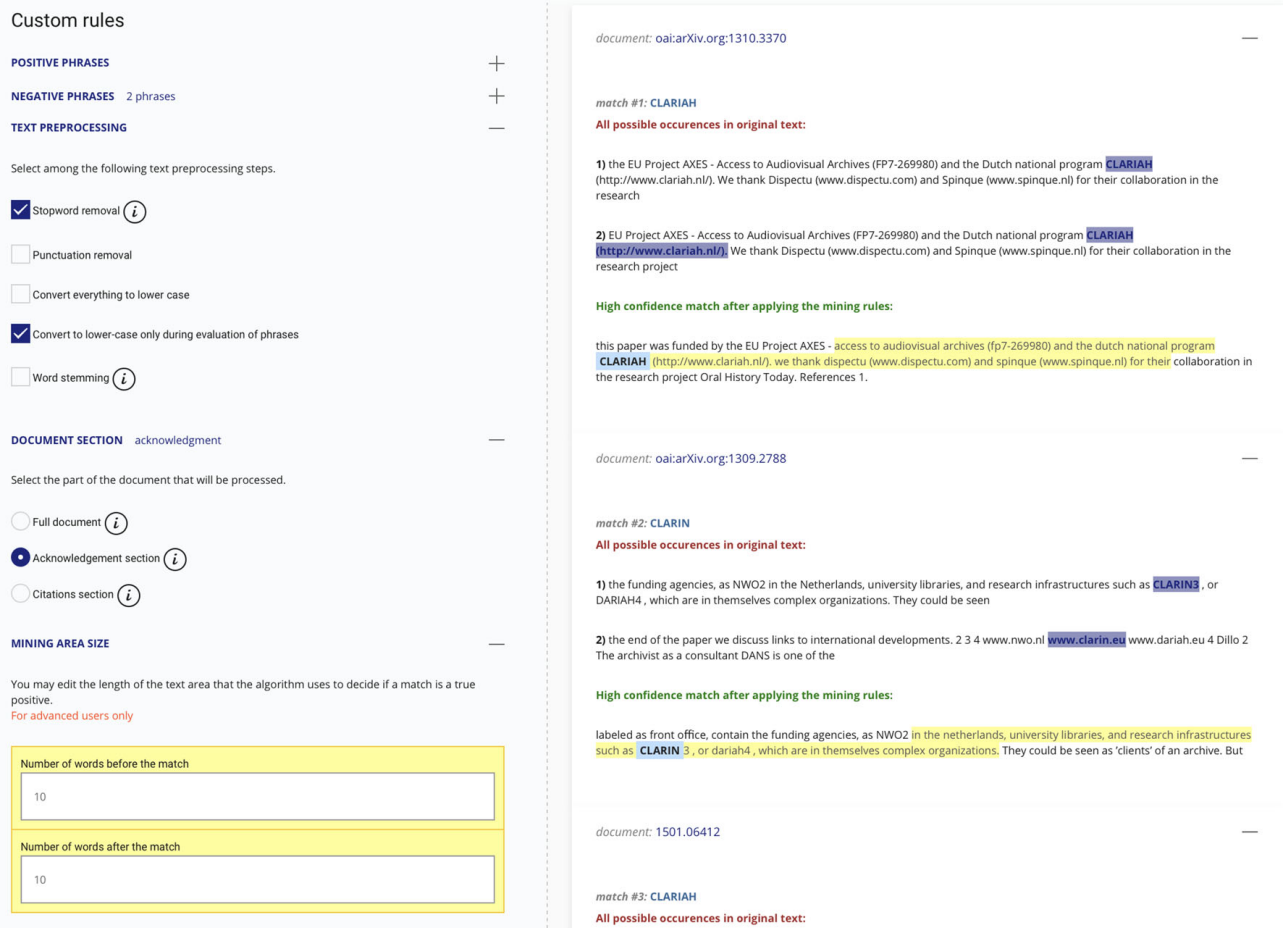
Information extraction interface


As already mentioned in the introduction, having a fully fledged text mining framework written in a declarative language (SQL) allows for easy implementation of application interfaces. An interactive information extraction interface on top of the presented library is deployed in OpenAIRE. Figure 1 depicts a screenshot of the interface. The purpose of the interface is to allow the users to implement and tune simple information extraction tasks. Specifically, they upload their documents in plain text format, and a CSV containing the entity titles/names for information extraction (i.e., in this example terms *clarin* and *clariah*^3^). Through this interface, they select preprocessing tasks (e.g., stopword removal, punctuation removal, and more), text extraction tasks (i.e., extract acknowledgement section, citation section), positive and negative terms and phrases to disambiguate false positives, and finally the length of the text snippet before and after an occurrence of the searched concept. The user selects the functions and mining rules using a graphical interface, and the SQL query is built in the backend and runs online returning the highlighted results to the domain expert who may modify her algorithm accordingly and rerun to obtain updated results. Using the rules as shown in the screenshot, the following query (Algorithm 5) is automatically produced:

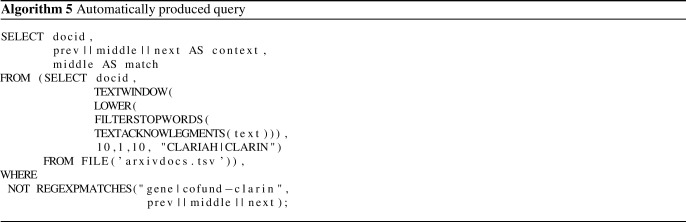


### Distributed deployment

DETEXA is integrated with YeSQL’s implementation on top of SQLite. Thus, each DETEXA instance runs single-threaded. Distributed deployment of DETEXA is applicable through its integration with popular state-of-the-art distributed systems. In OpenAIRE, DETEXA processes the data locally in each node of a Spark cluster. Specifically, Spark is responsible for the distribution and partitioning of data to multiple nodes. In each node, DETEXA is called as a Python command line executable, processes the data in standard input and outputs the results in JSON format in the standard output. The following command presents how DETEXA is launched in each node.
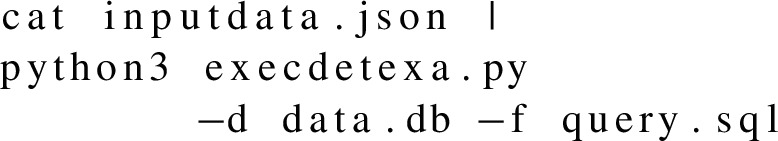


*data.db* is an SQLite database that contains supporting data for each specific text analysis task. *query.sql* contains the DETEXA query which runs the analysis. The output of this command is redirected to a file which contains the results in JSON.

## DETEXA in practice

DETEXA has been used by OpenAIRE data scientists to implement several scalable text mining workflows:

- *Citation matching algorithm* [12]. An algorithm for citation matching based on title matching that achieves higher recall and precision rates than the state-of-the-art, while running more than an order of magnitude faster. In particular, in OpenAIRE, we search for citations to datasets using their DOIs, titles, and other metadata (i.e., dates, creator names, publishers, etc.). We extract parts of the text which look like citations and search for datasets using database join and pattern-matching techniques. Based on the experiments described in the paper, the precision of the dataset extraction module is 98.5% and recall is 97.4% but it is also probably overestimated since it does not take into account corruptions that may take place during PDF-to-text extraction. It is calculated on the extracted full texts of small samples from PubMed and arXiv.
- *Funding information extraction*.^4^ An algorithm for extracting funding information from scientific papers using external data from the FundRef registry.^5^
- *Specific grant identifier linking (project mining)*. An algorithm for extracting funding information at the grant identifier (project) level from scientific publications using the OpenAIRE projects database. The mining algorithm works by utilizing (i) the grant identifier, and (ii) the project acronym (if available) of each project. The mining algorithm: (1) preprocesses/normalizes the full texts using several functions, which depend on the characteristics of each funder (i.e., the format of the grant identifiers), such as stopword and/or punctuation removal, tokenization, stemming, converting to lowercase; then (2) string matching of grant identifiers against the normalized text is done using database techniques; and (3) the results are validated and cleaned by looking at the context around the matched ID for relevant metadata and positive or negative words/phrases, in order to calculate a confidence value for each publication–project link. A confidence threshold is set to optimize high accuracy while minimizing false positives, such as matches with page or report numbers, post/zip codes, parts of telephone numbers, DOIs, URLs, or accession numbers. The algorithm also applies rules for disambiguating results, as different funders can share identical project IDs; for example, grant number 633172 could refer to the H2020 project EuroMix but also to Australian-funded NHMRC project ‘Brain activity (EEG) analysis and brain imaging techniques to measure the neurobiological effects of sleep apnea.’ Project mining works very well and was the first text and data mining (TDM) service of OpenAIRE. Performance results vary from funder to funder but precision rates are higher than 98% for all funders and 99.5% for EC FP7 and H2020 projects. Recall is higher than 95% (99% for EC projects) when projects are properly acknowledged using project/grant IDs. Note that this algorithm works by string matching of grant identifiers in the text (step 2), so without an ID present in a particular text, it cannot match any of the projects in our database. This occurred rarely in the collections which were studied. However, OpenAIRE includes a couple of funders without strong acknowledgement mandates, for which it is much more frequent for authors to acknowledge the funder only, omitting to state the specific project that funded their work. For such cases, we run an additional mining algorithm that can identify at the funder (rather than the project) level, linking to what we label an "unidentified" project.
- *Software mining* and linking to Software Heritage.^6^ This TDM module used in OpenAIRE runs also on parts of the text which look like citations. We search the citations for links to software in open software repositories, specifically GitHub, SourceForge, Bitbucket, and the Google Code archive. After that, we search for links that are included in Software Heritage (SH) and return the permanent URL that SH provides for each software project. We also enrich this content with user names, titles, and descriptions of the software projects, using web mining techniques. Since software mining is based on URL matching, our precision is 100% (we return a software link only if we find it in the text and there is no need to disambiguate). As for the recall rate, this is not calculable for this mining task. Although we apply all the necessary normalizations to the URLs in order to overcome usual issues (e.g., HTTP or HTTPS, existence of www or not, lower/upper case), we do not calculate cases where a piece of software is mentioned using its name and not by a link from the supported software repositories.
- *Communities mining*. Custom mining modules for linking research objects to specific research communities, initiatives, and infrastructures in OpenAIRE Connect,^7^ such as the mining module developed in early 2020 for the COVID-19 OpenAIRE Connect research community gateway,^8^ or the mining for the Connect gateway of the Digital Research Infrastructure for the Arts and Humanities (DARIAH).^9^
- *Bioentities extraction*. We have developed mining algorithms for identifying over 30 bioentities in publications’ plain texts which are currently in the process of being integrated into the OpenAIRE Research Graph.^10^ What has already been integrated are links to Protein Data Bank (PDB)^11^ entries [19]. We have downloaded the database with PDB codes and we update it regularly. We search through the whole publication’s full text for references to PDB codes. We apply disambiguation rules and contextual information so that we return valid results. PDB text mining is challenging since many PDB codes can be easily confused with other entities. For example, there are PDB codes that are the same as antibody codes, or that can be confused with dates (5DEC, 4NOV, 4MAY), numbers with exponents (6E00, 5E24, 4E08), time (6H20, 5MIN), or other measurements (5MHZ, 2GHZ, 1LUX, 4X20, 3MEN) and many more. The current precision is 98%. Although it is risky to mention recall rates since these are usually overestimated, we have calculated a recall rate of 98% using small samples from PubMed publications.
- *Interactive text analysis* [11]. An interactive mining platform which allows domain experts to define mining procedures, set/update mining rules, and validate the results, while the actual text mining code is produced automatically. This significantly reduces the communication between the developers and the experts and moreover allows the experts to experiment themselves using a user-friendly graphical interface (Fig. 1).
- ARIADNE and ARIADNEplus,^12^ archaeological data. In OpenAIRE’s collaboration with the H2020 EC project ARIADNEplus (A+), the aim is to link the A+ Data Infrastructure with repositories of scientific publications by exploiting OpenAIRE and the links to individual journals such as Internet Archaeology or Archaeology and Culture (A &C), in order to make archaeological data more discoverable, accessible, interconnected, and complete. A successful pilot study for building a bridge between ARIADNE and OpenAIRE during the first ARIADNE project utilized a text mining citation extraction & matching algorithm on ADS Grey Literature Reports that found almost 300 relations to OpenAIRE publications.^13^ In ARIADNEplus, this bridge is currently being built further by linking the project’s datasets with OpenAIRE: by first harvesting relevant dataset metadata from A+, we then text mine for publications that cite A+ data objects. Such relationships can then be used to link content between OpenAIRE and the ARIADNEplus portal,^14^ while also improving the configuration of the existing Digital Humanities and Cultural Heritage^15^ gateway in OpenAIRE.
- *Patent mining* and patent metadata enrichment through the Open Patent Services (OPS)^16^ API. This text mining module identifies EPO patents in the full text of publications of OpenAIRE, achieving a 90% precision accuracy.
- *Document classification* [10, 20–23]. Apart from text mining modules, OpenAIRE also provides a document classification service that employs analysis of free text stemming from the abstracts of the publications. The purpose of applying a document classification module is to assign to a scientific text one or more predefined content classes. In OpenAIRE, the currently used taxonomies are arXiv, MeSH (Medical Subject Headings), ACM, and DDC (Dewey Decimal Classification, or Dewey Decimal System).

### DETEXA limitations

DETEXA’s UDF library consists mainly of low-level functions which are used as building blocks to implement complex algorithms. A complex technique can be implemented using a combination of existing UDFs, SQL operators, and through the implementation of new custom UDFs as shown in Sect. 3.3. Currently, the DETEXA framework does not provide end-to-end implementations for modern techniques like BERT or more sophisticated semantic analysis. If those are to be used, then the developer has two options: (1) implement the techniques from scratch using DETEXA UDFs and SQL operators to utilize the performance enhancements of in-database analysis, or (2) register a polymorphic table-returning UDF which imports an external library that implements the desired functionalities. This is the fast way to use already existing code, e.g., a BERT classifier; however, this does not reap all the benefits of the underlying database engine (see also discussion in Sect. 3.1).

Note that DETEXA should not be considered as a framework that provides implementations of text mining algorithms, semantic analysis, and so on, but as an extensible framework that allows the scalable implementation of such techniques using a declarative language with a rich UDF library and an underlying data management system.

## Evaluation

### Experimental setup

Several experiments were conducted to compare the performance of end-to-end text analysis pipelines. We compared our framework against NLTK (v3.7), scikit-learn v(1.1) on CPython (v.3.8.10), and PySpark (v2.4.7). Our framework (DETEXA) is implemented on top of YeSQL’s integration with SQLite (v.3.31.11) and PyPy (v.7.3.6 with GCC 7.3.1) tracing JIT compiler. In our experiments, we selected three real word tasks of fundamental importance that have been gaining traction thanks to recent developments in the fields of text mining and natural language processing (NLP). The experiments ran on real publication abstracts from OpenAIRE. We ran all experiments on an Intel(R) Core i7-4790 processor with 3.60GHz and 4 cores/8 CPUs. The server has 16GB of main memory and runs Ubuntu 20.04. We executed all experiments with cold caches, and we report the average of 5 executions. DETEXA’s source code, queries, and datasets for the experiments are available at [7].

### Experiments

#### Project mining

We run an experiment to evaluate DETEXA’s performance in terms of execution time. We implemented an algorithm that mines research publications and extracts NSF (i.e., National Science Foundation) project identifiers. The last step of the algorithm is to calculate the count of funded publications per directorate.^17^ Authors acknowledge projects that funded their research in a specific acknowledgements section but also several times anywhere in the text or in footnotes. Thus, in this case, the algorithm processes the full texts. In this experiment, we compare against hand-optimized pure Python code as well as against an efficient data structure of Python (Pandas dataframe).
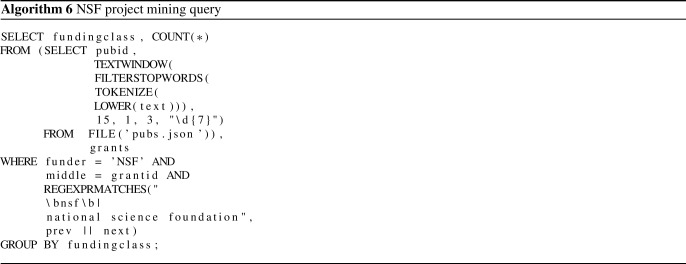


DETEXA query implementing NSF project mining query is Algorithm 6. The query reads data from two sources:

- An external file with publications’ full texts in JSON format with two keys: pubid, text. We used four versions of this file with various sizes.
- A table stored in the database including the project identifiers. This table includes the funder name, the grant agreement number, a unique project identifier, and the funding class (i.e., directorate in case of NSF). This table includes 467K NSF project identifiers as well as another $\sim$2 million project identifiers from other funders (e.g., EC, NIH, etc.)

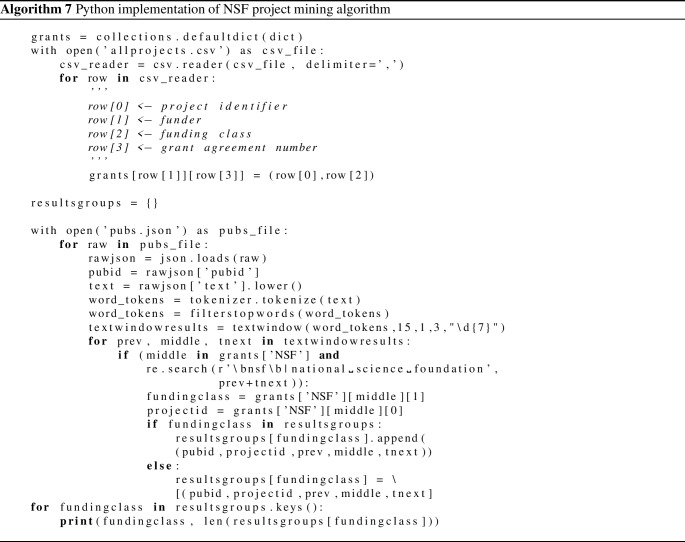


The query applies three preprocessing steps for each input text (i.e., text conversion to lowercase, tokenization with punctuation removal and stopword removal). Then, it scans the text and returns a rolling window. The window includes a middle word, as well as 15 words before the middle word and three words after the middle word. Since the NSF project identifiers are 7-digit numbers, the middle word is filtered with the appropriate pattern. Each middle word is joined against the NSF grant IDs from the grants table. The results are disambiguated with a pattern which filters out matches that do not contain strings ‘nsf’ or ‘national science foundation’ in their prev and next fields. Finally, the query groups the results by the fundingclass and counts the publications found.

The equivalent Python implementation is shown in Algorithm 7. Note that the implementation of functionalities textwindow, tokenize and filterstopwords is the same in all cases. In DETEXA, they are registered as UDFs, whereas in Python code they are imported and used directly in the code. As shown, the pure Python implementation requires a much larger and more cumbersome code. Specifically, the data loading, creation of appropriate data structures, the JOIN, and the GROUP BY, need to be implemented in procedural code. We used nested Python hash dictionaries to store the grant identifiers. The outer dictionary contains keys including the funder name. The inner dictionary’s keys include the grant identifiers and its values contain the unique project identifiers and the funding class. The selection of this struct is very important as it allows optimal execution of the JOIN between the middle field and the grant identifiers. A similar structure is used to implement the GROUP BY. In this case, the dictionary keys contain the extracted funding class. Obviously, if running a different analysis on the results, for example, a GROUP BY on a different column, we should apply several changes in the code. This does not happen in the case of DETEXA as it supports a declarative SQL language which allows easy modifications. Last but not least, implementing such an analysis in Python requires careful selection of the appropriate structures, whereas in a declarative language on top of a DBMS, the DBMS itself and the query optimizer decide the optimal way to process a relational operator using advanced data structures to execute a JOIN or a GROUP BY.

**Fig. 2:**
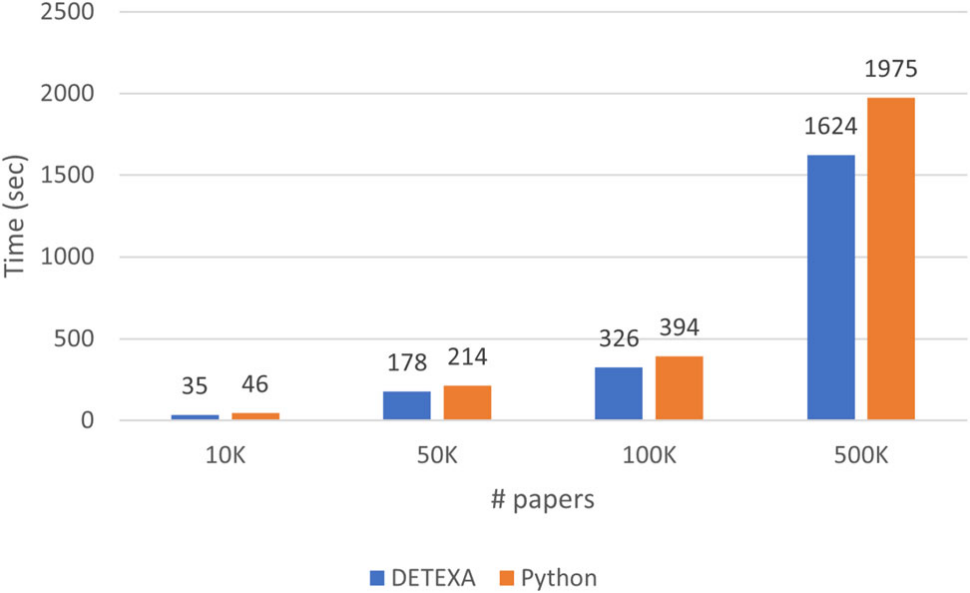
Execution times of DETEXA and Python for NSF project mining

Figure 2 shows the execution times of the DETEXA and the pure Python implementation for different sizes (i.e., 10K, 50K, 100K, and 500K documents) of input data. In this experiment, the grants file contains projects from 22 funders, thus in Python, the outer dict contains 22 entries. There are also 467K NSF 7-digit IDs ($\sim$3MBs) that participate in the actual JOIN. The mining results consist of 9345 links to projects in the larger dataset. The final GROUP BY produces eight groups where each group corresponds to a unique directorate. These counts are small enough so that Python’s dictionaries can handle them very well and clearly the main overhead of the execution is in the preprocessing steps on the publication’s full texts which is done using the same code in both cases. Still, as shown, DETEXA outperforms the hand-optimized Python implementation in all different data sizes in this experiment. Interestingly, if we make the wrong decision and use Python lists to load the grant identifiers, DETEXA runs 48 times faster than Python, which proves our claim that using procedural Python code for such tasks passes to the developer the responsibility of making the important decision of selecting the appropriate structures.

We also ran the same experiment using an implementation with Pandas dataframes. Pandas is a powerful in-memory tool with several functionalities with support for string attributes. However, it offers limited native functionalities for text processing. For example, it does not support stopword removal and other complex functions which should be first implemented outside Pandas and then applied on Pandas dataframes or series. Since the Pandas library is written in C, execution of custom functions in Python involves significant overheads (i.e., data copies, data types transformations, context switches between Python and C) which results in much slower execution than DETEXA and hand-optimized Python code. Specifically, the execution times for Pandas implementation are 116 and 623 s, for 10K and 50K documents respectively. For the 50K dataset, preprocessing of documents which uses custom functions implemented outside Pandas took 562 s in total. DETEXA runs more than three times faster than Pandas (35 and 178 s).

Furthermore, a Pandas dataframe fails with data larger than memory, passing to the developer the responsibility to handle such cases with more complex code using chunks. For example, in our experiment, a single Pandas dataframe cannot handle the 500K pubs dataset of this experiment and fails with memory error.

#### Document classification

The next experiment is a text classification task [10], defined as assigning predefined category labels to new papers based on the likelihood suggested by a training set of labeled papers. Input data for text classification consist of raw, unstructured text. Before feeding input data to any classifier, it is projected into an appropriate feature space by applying preprocessing procedures which transform plain texts into lists of terms (keywords, metadata). Terms may be single words or n-grams. Since text classification is a supervised learning task, it has two main phases: (i) the training phase, in which a global list of unique terms is updated, along with the respective term frequencies for each paper; and (ii) the prediction phase, in which the classifier predicts the labels of a given paper based on its content (list of terms).

In our experiments, we assume that the training phase has already been executed. The prediction phase of the algorithm involves three preprocessing operators running sequentially. These are tokenization, stopword removal, and stemming. Terms may be single words or 2-grams. We have implemented the classification sub-module in the presented DETEXA framework which is hand-optimized with Python code as well as in NLTK. The DETEXA query implementing the classification sub-module was shown in the introduction.

**Fig. 3:**
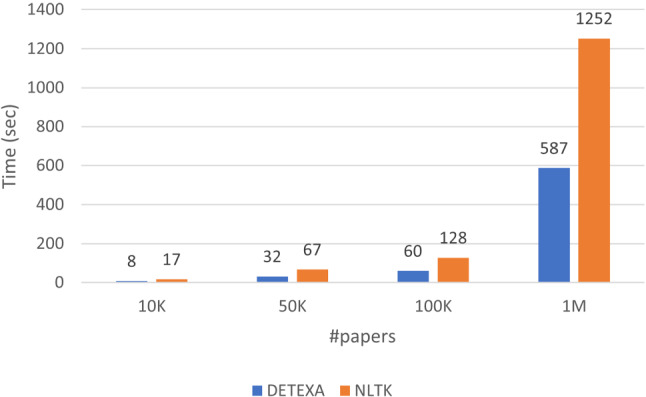
Execution times of DETEXA and NLTK for the document classification task


We ran this experiment using 10K, 50K, 100K, and 1 M text abstracts. Figure 3 shows the execution times for the classification sub-module using our presented framework compared with NTKL for the different sizes of input data. In all cases, the time needed for the execution using DETEXA falls in half. This happens for 2 reasons: (1) the design of the framework which effectively maps text analysis tasks in database UDFs and lets the DBMS handle the heavy relational tasks (e.g., JOINs, GROUP BY’s), and (2) the performance characteristics which are inherited by YeSQL.

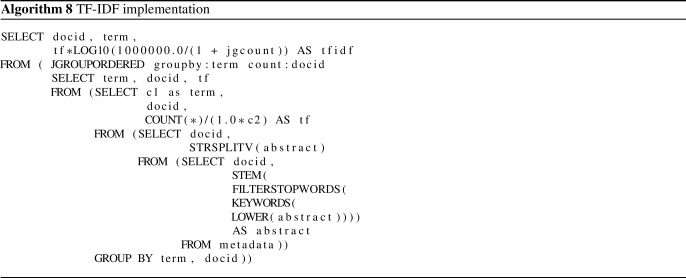


#### Term frequency-inverse document frequency (TF-IDF)

The third task is the implementation of the TF-IDF algorithm. TF-IDF is a predominant feature vectorization method widely used in many NLP applications, as well as in text classification and text mining systems. For example, search engines use TF-IDF to rank the relevance of a document for a query. TF-IDF is also used to recommend papers to authors or to detect similarities among papers. TF-IDF is divided into two parts: computation of TF (term frequency) and computation of IDF (inverse document frequency). TF is the number of times a term appears in a document divided by the total number of words in the document. IDF of a term reflects the proportion of documents in the corpus that contain the term: it is defined as the logarithmic fraction obtained by dividing the total number of documents in the corpus by the number of documents in the corpus containing the term. TF-IDF of a term is computed by multiplying TF and IDF scores.

We have implemented the TF-IDF algorithm in the presented DETEXA framework, as well as in a hand-optimized Python implementation using NLTK. We have also used the implementation of scikit-learn from [24] that uses the native *TfidfVectorizer* sklearn package. The DETEXA query implementing the TF-IDF task is shown in Algorithm 8.

This query reads data from a table that contains publication abstracts. First, it executes three preprocessing operators running one after another. These are tokenization with punctuation removal, stopword removal, and stemming. With the dynamic function STRSPLITV, the input string with stemmed words is split vertically into a database column, and a new column containing the number of stemmed words in each abstract is added. Then, using relational operations, the TF score is calculated. Since the heavy data processing task (i.e., GROUP BY) has been processed by the DBMS, JGROUPORDERED UDF joins consecutive rows of the table having the same term and counts the number of documents in the corpus containing the term at hand. That is of high importance, as we do not need to execute another database GROUP BY operator with an aggregate function, which is a heavy data processing task; we exploit the fact that the intermediate data is already sorted by ‘term’ column during the previous GROUP BY. Finally, the query calculates *tfidf* score.

**Fig. 4:**
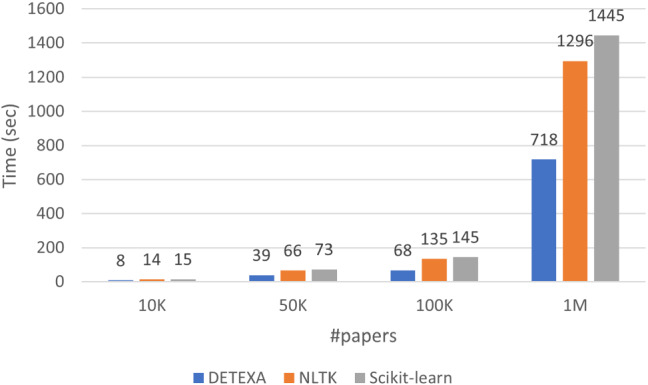
Execution times of DETEXA, NLTK, Scikit-learn for TF-IDF

We ran the experiment using 10K, 50K, 100K, and 1 M text abstracts. Figure 4 shows the execution times for the TF-IDF task using our framework, as well as NLTK and scikit-learn. The results compared to NLTK reveal very similar trends to the previous experiment. Furthermore, using the proposed framework, the TF-IDF algorithm runs much faster than the native implementation of scikit-learn.

**Fig. 5:**
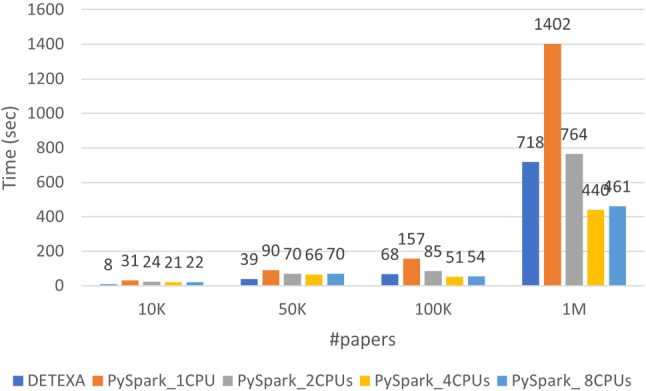
Execution times of DETEXA and Spark (various parallelisms) for TF-IDF

Finally, we ran a separate experiment on TF-IDF to compare the DETEXA framework with PySpark and its native implementation of *tfidf* (*HashingTF*, *IDF* packages). We selected PySpark, since it is a popular distributed analytical system which achieves efficient execution of algorithms through parallelization. YeSQL’s implementation on top of SQLite runs only single-threaded. However, we compared it against PySpark with different configurations (i.e., single CPU execution, 2 CPUs, 4 CPUs, and 8 CPUs). As shown in Fig. 5, implementing TF-IDF with the proposed UDF library runs faster than Spark, not only in single-threaded mode but also if Spark runs using 2 CPUs in all data sizes. Spark achieves its best performance with 4 CPUs, where it runs faster than our single-core solution in 100 K and 1 M abstracts. This result indicates a potentially interesting future direction and testing as well: deployment of the proposed framework with multi-threaded DBMSs (e.g., MonetDB is also supported by YeSQL) will reap the benefits of parallel execution.

## Conclusions

We presented an efficient and extensible text analysis framework built on top of YeSQL. The DETEXA framework maps several text mining functionalities to reusable scalar, aggregate, and polymorphic table UDFs written in Python. Due to the performance characteristics of YeSQL and its design, the presented framework is able to execute faster than other popular solutions critical text mining analytical tasks. The declarative language of the DETEXA framework allows fast experimentation and implementation, as well as easy development of application interfaces. We actively concentrate on various future directions, including integration of the framework with other DBMSs that are supported by YeSQL in order to reap the benefits of multi-threaded/parallel execution, and richer interfaces to offer the opportunity to users without programming knowledge to implement and tune complex text analysis tasks. Implementation of modern techniques like foundation models and sophisticated semantic analysis within DETEXA is also in our future plans.

## Footnotes

1 https://www.openaire.eu, https://explore.openaire.eu.

2 https://github.com/openaire/iis.

3 https://www.clarin.eu/.

4 http://opensciencefair.eu/posters/funding-information-extraction.

5 https://www.crossref.org/services/funder-registry/.

6 https://www.softwareheritage.org.

7 https://connect.openaire.eu.

8 https://covid-19.openaire.eu.

9 https://dariah.openaire.eu.

10 https://graph.openaire.eu.

11 https://www.rcsb.org.

12 https://ariadne-infrastructure.eu.

13 https://www.openaire.eu/ariadne-openaire-collaboration.

14 https://portal.ariadne-infrastructure.eu.

15 https://dh-ch.openaire.eu.

16 https://www.epo.org/searching-for-patents/data/web-services/ops.html.

17 https://www.nsf.gov/staff/orglist.jsp.
